# Why does the hemolytic activity of silica predict its pro-inflammatory activity?

**DOI:** 10.1186/s12989-014-0076-y

**Published:** 2014-12-19

**Authors:** Cristina Pavan, Virginie Rabolli, Maura Tomatis, Bice Fubini, Dominique Lison

**Affiliations:** Department of Chemistry, “G. Scansetti” Interdepartmental Center for Studies on Asbestos and Other Toxic Particulates, University of Torino, Via P. Giuria 7, 10125 Turin, Italy; Louvain Center for Toxicology and Applied Pharmacology (LTAP), Université catholique de Louvain, Avenue E. Mounier 52 – bte B1.52.12, 1200 Brussels, Belgium

**Keywords:** Red blood cells, Hemolysis, Inflammasome, Silica, Quartz, IL-1β, Lysosomal damage, Phagolysosome, Alveolar macrophages, Membrane interaction

## Abstract

**Background:**

The hemolytic activity of inhaled particles such as silica has been widely investigated in the past and represents a usual toxicological endpoint to characterize particle reactivity despite the fact that red blood cells (RBCs) are not involved in the pathogenesis of pulmonary inflammation or fibrosis caused by some inhaled particles. The inflammatory process induced by silica starts with the activation of the inflammasome, which leads to the release of mature IL-1β. One of the upstream mechanisms causing activation of the inflammasome is the labilization of the phagolysosomal membrane after particle phagocytosis. Considering RBC lysis as a model of membrane damage, we evaluated the relationship between hemolytic activity and inflammasome-dependent release of IL-1β for a panel of selected silica particles, in search of the toxicological significance of the hemolytic activity of an inhaled particle.

**Methods:**

Well-characterized silica particles, including four quartz samples and a vitreous silica, with different surface properties and hemolytic potential were tested for their capacity to induce inflammasome-dependent release of IL-1β in LPS-primed primary murine peritoneal macrophages by ELISA and Western blot analysis. The mechanisms of IL-1β maturation and release were clarified by using ASC-deficient cells and inhibitors of phagocytosis and cathepsin B.

**Results:**

The silica samples induced dose-dependent hemolysis and IL-1β release of different amplitudes. A significant correlation between IL-1β release and hemolytic activity was evidenced (r = 0.827) by linear regression analysis. IL-1β release was completely abolished in ASC-deficient cells and reduced by inhibitors, confirming the involvement of the inflammasome and the requirement of phagocytosis and cathepsin B for activation.

**Conclusions:**

The same physico-chemical properties of silica particles which are relevant for the lysis of the RBC membrane also appear implicated in the labilization of the phagolysosome, leading to inflammasome activation and release of the pro-inflammatory cytokine IL-1β. These findings strengthen the relevance of the hemolysis assay to predict the pro-inflammatory activity of silica dusts.

**Electronic supplementary material:**

The online version of this article (doi:10.1186/s12989-014-0076-y) contains supplementary material, which is available to authorized users.

## Background

Even if the pathogenicity of silica particles is known from ancient times, it remains one of the most puzzling issues of particle toxicology [1]. The mechanism of action of crystalline silica dusts was deeply investigated in the 50′, when the incidence of silicosis caused by exposure to respirable dusts was high in numerous occupational settings; then revisited by many investigators by the end of the 90′ because of the progressive awareness that, under some circumstances, crystalline silica is also a human carcinogen [2]. The potential toxicity of silica is today back to the stage with the growing interest in nanotechnology and the use of amorphous silica nanoparticles (NPs) for several applications, including biomedicine. A large number of studies highlighted the role of lung cells (e.g. macrophages, epithelial cells) in the development of silica-induced diseases, but the physico-chemical properties of silica particles determining these cellular responses and the overall mechanism of toxicity remains only partially solved. Indeed, both *in vivo* and *in vitro* studies reflect a great variety of cellular responses to silica, not only among various forms (for instance, crystalline silica is known to cause chronic effects such as silicosis and cancer while amorphous silica produces only transient inflammation) and polymorphs of silica, but also among quartz specimens of similar origin [3,4]. Differences found in the carcinogenic activity in humans [5,6] and in experimental studies [7] led to the awareness of a “variability” of silica hazard, ascribed to the multiplicity of the physico-chemical properties of silica involved and to differences in the surface state of apparently similar silica samples [8].

Although red blood cells (RBCs) do not play any role in the inflammatory or fibrotic responses induced by silica, the RBC membrane has been traditionally regarded as a simple and convenient experimental model [9] and the hemolysis test was largely used in the past to assess the surface reactivity of silica and many more mineral species [9-11]. More recently, a number of studies have re-considered the hemolytic activity of inorganic particles, especially of silica [12-18]. In spite of conflicting opinions on the consistency between *in vitro* and *in vivo* studies due to a plethora of measurable endpoints for each toxicological manifestation [11,19], the hemolytic activity has been considered one of the best predictors of *in vivo* inflammation for metal oxide nanoparticles [14,18]. In particular, a correlation between the ability of some quartz dusts to cause *in vivo* inflammation and to induce hemolysis *in vitro* was found, supporting the contention that lung inflammogenicity is driven by some surface properties of quartz [12,13]. However, a link between hemolytic activity and cellular responses, e.g. cytotoxicity, has not always been found because of the complexity of the physico-chemical determinants imparting toxicity to a silica particle. Each property may be differently involved in the various steps of the pathogenic response to silica [8] and could differently affect each cell type [16]. All these contrasting findings leave open the question about the toxicological significance of assessing the hemolytic activity of an inhaled particle. Silica particles are highly hemolytic and - as with other silica-related biological responses - the hemolytic activity also varies dramatically from one to the other silica specimens in a rather complex way. In a previous study we have used a large set of silica samples, differing in most of the physico-chemical properties claimed to be related to cellular responses to silica, in order to identify which was the major feature determining RBC lysis. Hemolytic activity varied from absent to very high. From a detailed analysis and comparison, it was concluded that the surface distribution of silanols, silanolate and siloxane was the primary factor causing hemolysis [20]. Taking advantage of the availability of this panel of well-characterized silica samples largely differing in their hemolytic potential, we have here attempted to find a relationship between silica hemolytic activity and the reported biological events involved in the progression towards silicosis and cancer [6].

The inflammatory reaction is one of the first steps involved in the lung pathogenesis induced by silica. Recent reports revealed that both crystalline [21-23] and amorphous silica [24-26], trigger inflammation through the activation of the inflammasome protein complex which regulates the maturation and release of cytokines of the IL-1 family [27]. The Nalp3 receptor (also known as NLRP3), member of a family of cytoplasmic immune receptors (NLRs), is involved in this reaction. When activated, Nalp3 can recruit the adaptor apoptosis-associated speck-like protein (ASC) inducing the activation of the proteolytic enzyme caspase-1. The latter initiates cell death and controls the cleavage and secretion of the pro-inflammatory cytokine interleukin IL-1β [28,29] whose persistent overproduction has been linked to silicosis [30]. The upstream biochemical mechanism of Nalp3 inflammasome activation is still partially unclear [31,32], but two pathways, probably interconnected, have been proposed [33]. The first one involves ROS generation, that could activate directly or indirectly Nalp3 [21,22,34,35], the second entails lysosome damage leading to release the lysosomal content, including hydrolytic enzymes such as cathepsin B, into the cytosol. This hypothesis is based on the observation that both phagosomal destabilization induced by particles [23,24,36,37] and pharmacological disruption of lysosomes [23] lead to the activation of the Nalp3 inflammasome. Early research in the past century had already revealed the ability of alveolar macrophages to incorporate insoluble particles into a phagolysosome, thus initiating cell death pathways following disruption of the phagolysosome [38-40].

Since damage to the phagolysosome is a crucial event in triggering the inflammatory pathway caused by silica particles, we hypothesized here that direct interaction of the lysosomal membrane with specific functionalities on silica particle surface (e.g. silanol groups, silanolates, siloxanes) plays a role in lysosomal destabilization similarly to the way they cause the lysis of RBCs. The aim of the present study is to investigate the pro-inflammatory response by measuring inflammasome activation by a panel of silica samples selected for their diverse RBC lysis activity, and to evaluate the relationship between their capacity to activate the inflammasome and their hemolytic activity. In order to span a large interval in hemolytic potential, we have chosen two very active silica samples, two with intermediate activity and an inactive one. Release of IL-1β was assessed in primary murine peritoneal macrophages. To verify the role of the inflammasome in IL-1β release, experiments were performed in macrophages from ASC-deficient mice *versus* wild-type. To clarify the mechanism leading to inflammasome activation, the experiments were repeated in the presence of an inhibitor of phagocytosis and of the lysosomal enzyme cathepsin B.

## Results

### Physico-chemical properties and hemolytic potential of the silica samples investigated

The study was conducted with the following five samples:

1. the commercial microcrystalline α-quartz Min-U-Sil 5 (*Qz-1*);
2. Min-U-Sil 5 heated at 800°C (*Qz-2*);
3. a pure quartz obtained by grinding a crystal from Madagascar (*Qz-3*);
4. a pure ground quartz etched with hydrofluoric acid (*Qz-4*);
5. a vitreous silica obtained by grinding a very pure silica glass (*VS*).

The physico-chemical properties of the silica particles are reported in Table 1. All the samples considered were obtained by grinding. Their morphology is thus irregular, with acute spikes and edges, typical of ground material. Except vitreous silica (VS), they all have a crystalline structure. They were selected for the present investigation because of their similar size and surface area but different hemolytic activity and silanol distribution. We have also shown that the hemolytic activity of these samples is unrelated to their potential to release free radicals [20], and Qz-3, Qz-4 and VS exhibited a limited potential to generate particle-derived free radicals [41], which was appropriate to minimize other factors than membranolytic activity that may activate the inflammasome.

**Table 1:** **Main physico-chemical characteristics and hemolytic activity of the selected silica samples**

**Silica**	**SSA (m** ^**2**^ **/g)** ^**b**^	**Particle size (μm)** ^**c**^		**Major metal impurities (% oxides)**	**Free radical generation** ^**d**^		**% Hemolysis (at 100 cm** ^**2**^ **/ml)**	**Ref.**
		**Average diameter**	**90% percentile**		**HO˙**	**COO˙ˉ**		
Qz-1	5.2	1.7 ± 0.7	2.6	Al 1.4, Fe 0.06	++	+++	++^a^	[20,47]
Qz-2	5.2	1.4 ± 0.6	2.1	Nd	Nd	Nd	+^a^	[20]
Qz-3	6.1	1.7 ± 1.8	2.8	Absent	++	absent	absent	[41]
Qz-4	6.1	1.5 ± 1.0	2.5	Nd	Nd	Nd	+^a^	-
VS	3.1	1.6 ± 1.2	2.7	Absent	+	absent	++^a^	[20,40]

^a^+, 10-20%; ++, 35-45% hemolysis.

^b^Specific surface area (SSA) evaluated by BET (Brunauer, Emmet and Teller method).

^c^Determined by flow particle image analysis which measures the average diameter expressed as circle equivalent (CE) diameter ± SD. The 90% percentile is the value of the CE diameter below which 90% of observations falls.

^d^Measured by electron paramagnetic resonance (EPR) spectroscopy and using DMPO as a trapping agent. Hydrogen peroxide or sodium formate were used as target molecules to generate respectively hydroxyl (HO˙) or carboxyl (COO˙ˉ) radicals.

Nd: Not determined.

**Figure 1:**
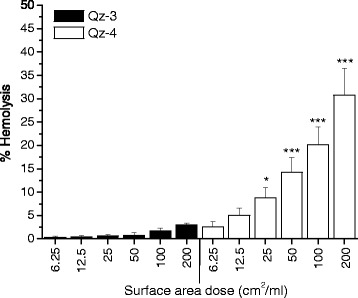
**Hemolytic activity of Qz-3 (pure quartz) and Qz-4 (pure quartz etched with HF).** Qz-3 and Qz-4 were incubated at increasing concentrations expressed as surface area doses (cm^2^/ml) in the presence of human red blood cells. Values are mean ± SD from five independent experiments. *p < 0.05 and ***p < 0.001 *vs* control not exposed to silica particles.

We previously reported the hemolytic potential of Qz-1, Qz-2 and VS [20] (*Qz-1 ≅ VS* > *Qz-2)*. The RBC lysis activity of Qz-3 (originating from a different batch than that previously tested in [20]) and Qz-4 (not tested before) was examined here (Figure 1) and compared in Table 1 with the hemolytic activity of the other three silica samples. Since RBC membranolysis is a surface-driven process, doses were expressed per surface area unit (Table 1). Both silica samples showed a dose-dependent hemolytic activity from 6.25 up to 200 cm^2^/ml. The hemolytic potential of Qz-4 was higher than that of Qz-3 at any of the doses investigated.

### Varying cytotoxic responses of murine macrophages

**Figure 2:**
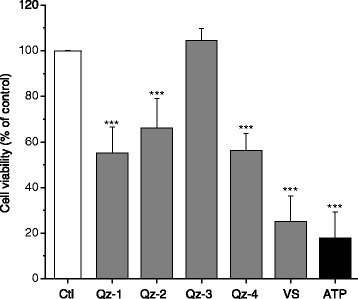
**Cytotoxic activity of different silica particles in primary murine macrophages.** LPS-primed primary murine macrophages were incubated with 20 cm^2^/ml of silica for 6 h and then evaluated for cell viability by means of the WST-1 assay. The silica samples included a commercial quartz (Qz-1), the same quartz heated at 800°C (Qz-2), a vitreous silica (VS), a pure quartz (Qz-3) and the pure quartz etched with HF (Qz-4). ATP (5 mM) was used as positive control. Results are expressed as percentage of the control (macrophages not exposed to silica particles -Ctl). Values are mean ± SD from at least three independent experiments. ***p < 0.001 *vs* control not exposed to silica particles.

Cytotoxic activity was determined in preliminary experiments over a range of concentrations (Additional file 1: Figure S1) in order to avoid the use of cytotoxic doses and irrelevant release of immature pro-IL-1β in the culture supernatant in subsequent experiments. Primary murine peritoneal macrophages were primed with LPS [22] and incubated 6 h with LPS-free silica particles. Figure 2 shows the cell viability determined by WST-1 assay at the concentration of 20 cm^2^/ml of silica sample. VS was the most cytotoxic, followed by the commercial quartz Qz-1 and Qz-2. Qz-3 was not statistically different compared to the control, while Qz-4 showed an intermediate cytotoxic activity. Only VS was already cytotoxic at the lowest dose of 10 cm^2^/ml. Qz-2 was less cytotoxic than Qz-1 at the highest dose of 40 cm^2^/ml, while Qz-3 was fully inactive at all the doses investigated (Additional file 1: Figure S1). Based on these results, we selected a low (20 cm^2^/ml) and a moderately cytotoxic dose (40 cm^2^/ml) as appropriate to evaluate silica-induced mature IL-1β release.

### Varying activation of IL-1β release

**Figure 3:**
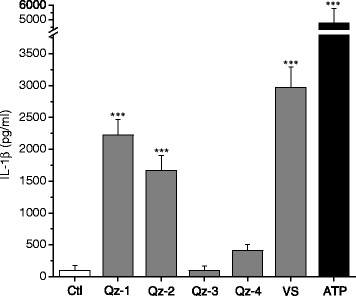
**IL-1β release from primary murine macrophages induced by different silica particles.** LPS-primed primary murine macrophages were incubated with 20 cm^2^/ml of silica for 6 h and then evaluated for IL-1β production (pg/ml) in culture supernatants by ELISA. The silica samples were a commercial quartz (Qz-1), the same heated at 800°C (Qz-2), a vitreous silica (VS), a pure quartz (Qz-3) and the pure quartz etched with HF (Qz-4). ATP (5 mM) was used as positive control. Values are mean ± SD including data from three independent experiments. ***p < 0.001 *vs* control not exposed to silica particles.

The effects caused by the set of silica particles on IL-1β release from primary murine peritoneal macrophages are reported in Figure 3. After 6 h incubation with the particles at the concentration of 20 cm^2^/ml, culture supernatants were collected to determine the levels of IL-1β produced. A large variation in the capacity to activate IL-1β release from one to the other silica particle was detected. Significant levels of IL-1β were induced by all the silica samples, with the exception of Qz-3. The latter was also tested at the highest dose of 40 cm^2^/ml and remained inactive (Additional file 1: Figure S2). Qz-1 and Qz-2 induced high level of IL-1β secretion (increased at 40 cm^2^/ml), while IL-1β release was definitely high for VS at both concentrations. Qz-4 induced a low release of IL-1β, more than Qz-3 but less than all the other silica samples. On the basis of these results, subsequent experiments on IL-1β release were carried out at 20 cm^2^/ml.

### Release of mature IL-1β is inflammasome-dependent and requires phagocytosis and lysosomal rupture

**Figure 4:**
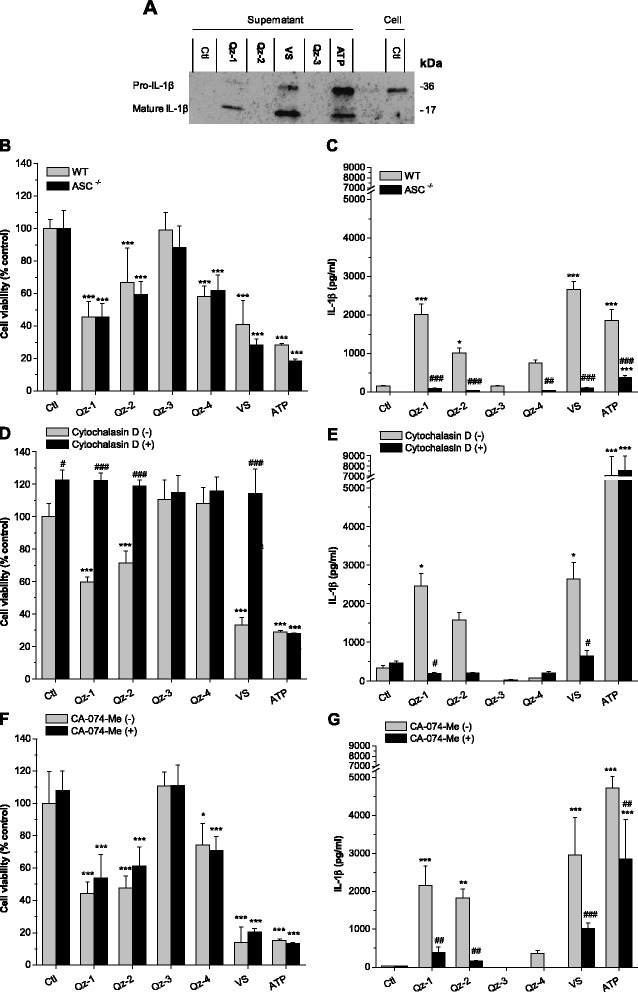
**Involvement of the inflammasome, phagocytosis and cathepsin B in inducing the release of mature IL-1β by different silica particles.** LPS-primed primary murine macrophages were incubated with silica at equal surface dose (20 cm^2^/ml) and with ATP (5 mM) as positive control. Culture supernatants were collected after 6 h (1 h for ATP) and cell viability by WST-1 **(B, D, F)** and IL-1β release (pg/ml) by ELISA **(C, E, G)** were measured. **(A)** A representative Western blot analysis conducted on culture supernatants or cell extracts to detect pro-IL-1β (17 kDa) or mature IL-1β (36 kDa) is shown. **(B, C)** WT mice and mice deficient in the adaptor molecule ASC (ASC^−/−^) are compared. Determinations were performed in six replicates **(B)** or quadruplicate **(C)** in a single experiment and expressed as the mean ± SD. Cells pre-treated for 1 h with cytochalasin D (5 μM) **(D, E)** or CA-074-Me (10 μM) **(F, G)** are compared to untreated cells. Determinations were performed in quadruplicate and expressed as the mean ± SD. Data from one representative experiment out of two **(D, E)** or one single experiment **(F, G)** are depicted. *p < 0.05, **p < 0.01 and ***p < 0.001 *vs* control not exposed to silica particles in each group; ^#^p < 0.05, ^##^p < 0.01 and ^###^p < 0.001 WT *vs* ASC^−/−^ or non-treated *vs* treated with inhibitors, for each sample.

To discriminate between mature IL-1β released into the supernatant after inflammasome activation and immature pro-IL-1β potentially due to silica cytotoxicity, we performed a Western blot analysis to separate the two proteins on the basis of their different molecular weight. LPS-primed macrophages were incubated for 6 h with 20 cm^2^/ml of silica or with ATP (5 mM) used as positive control. As shown in Figure 4A, significant differences in the expression levels of mature IL-1β among cells treated with various types of silica were observed. VS showed the highest amount of mature IL-1β, even more than the ATP positive control. Qz-1 induced a lower amount of mature IL-1β compared to VS, whereas IL-1β could not be detected for Qz-2 and Qz-3. Detection of mature/pro-IL-1β in the supernatant or cell extract not exposed to silica (Ctl) showed that priming with LPS was effective because only the pro-IL-1β form was present in cell extract, and no release of mature IL-1β was found in the supernatant. These results are consistent with the data obtained by ELISA. In order to evaluate the involvement of inflammasome in the release of IL-1β by several silica types and to confirm the mechanism leading to its activation, ASC-deficient cells, a phagocytosis inhibitor and an inhibitor of cathepsin B were used. Peritoneal macrophages from mice knock-out for the apoptosis-associated speck-like protein (ASC^−/−^), which is one of the three main components of the inflammasome complex, were compared with macrophages from wild type mice (WT, C57BL/6). No significant difference in cytotoxic activity between WT and ASC^−/−^ macrophages was found (Figure 4B), indicating that silica-induced cytotoxicity is independent from ASC, as reported by Cassel et al. for Nalp3 [22]. LPS-primed ASC^−/−^ macrophages displayed an evident defect in their ability to induce IL-1β compared with macrophages from WT (Figure 4C). The relative order of cytotoxicity and IL-1β production of the silica samples reflected the one already reported in Figures 2 and 3.

To inhibit phagocytosis, macrophages were pre-treated with cytochalasin D, an inhibitor of actin filament polymerization. Cytotoxicity was clearly reduced in the presence of cytochalasin D (Figure 4D). Cytochalasin D also reduced silica-induced IL-1β secretion (Figure 4E), whereas neither cytotoxicity nor IL-1β release were affected in cells challenged with ATP, a non-particulate inflammasome activator that does not require cellular phagocytosis. To investigate whether cathepsin B, an hydrolytic enzyme released into the cytosol after lysosomal destabilization, was involved in silica-induced IL-1β production [23], cells were pre-treated with a membrane-permeable cathepsin B-specific inhibitor (CA-074-Me). Cell toxicity was not affected by the inhibitor (Figure 4F), while IL-1β response was markedly reduced for all the silicas (Figure 4G). Overall, the present results indicate that cytotoxicity is induced after internalization of the particles, and that particle uptake and the ASC protein are required for IL-1β processing in macrophages for all the silica samples tested. Moreover, silica-induced IL-1β production is triggered by active cathepsin B present into the cytoplasm, suggesting that lysosomal damage, leakage of lysosomal active enzymes into the cytosol and finally activation of the inflammasome occurred [22,23,36].

### The release of IL-1β induced by the different silica samples correlates with their hemolytic activity

**Figure 5:**
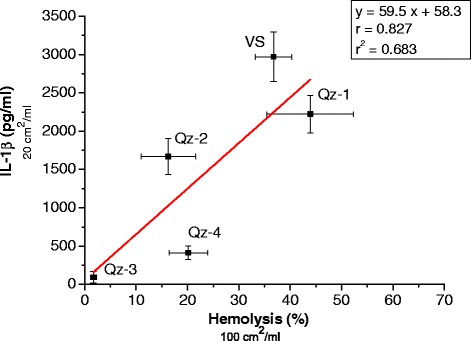
**Correlation between hemolytic activity and IL-1β release caused by silica particles.** Percent of hemolysis at silica concentration of 100 cm^2^/ml and release of IL-1β (pg/ml) from murine primary macrophages at silica concentration of 20 cm^2^/ml were compared by linear regression analysis. Values for hemolysis (%) are means of three to five independent experiments, while values for IL-1β (pg/mL) are means ± SD including data from three independent experiments. Parametric linear regression analysis (Pearson) was applied.

IL-1β levels induced by the different types of silica were reported as a function of their hemolytic activity in Figure 5. A linear regression analysis between the hemolytic activity and IL-1β release from primary murine macrophages indicates a clear correlation (r = 0.827) for the panel of silica particles here investigated.

## Discussion

The physico-chemical properties of silica may play different and specific roles in initiating the cascade of events resulting in the inflammatory and fibrotic responses involved in silicosis. A tentative association between the surface properties of silica particles and the sequence of events leading to the pathogenic condition after *in vivo* inhalation has been proposed by Fubini [8]. Among the various surface functionalities present on silica, some are related to particle-membrane interactions, such as the response observed in the hemolytic assay, and others to the activation of lung cells (e.g. alveolar macrophages and polymorphonucleated cells) which secrete inflammatory mediators and lead to development of inflammation and fibrosis. It is still not clear however, how these properties and the biological responses are interconnected.

All the silica samples examined here, except the non-hemolytic quartz (Qz-3), induced a pro-inflammatory effect promoting secretion of IL-1β. This response was highly varying among the set of selected silica particles, which reveals that variations are mostly due to the surface properties of the particles as sizes and surface areas were very similar. Min-U-Sil 5 quartz heated under drastic conditions (Qz-2), with the aim to reduce surface hydrophilicity by silanol condensation [42,43], was less active for IL-1β release than non-treated Min-U-Sil 5 (Qz-1). The pure quartz etched with hydrofluoric acid (Qz-4) to dissolve the external amorphous layer and remove surface defects [44] was more active than the pure Qz-3 in inducing a pro-inflammatory response. Vitreous silica (VS), with physico-chemical features very similar to a quartz dust except crystallinity [41], was even more inflammogenic than all quartz. This last point indicates that crystallinity is not required to produce an IL-1β response to silica, as previously reported by Sandberg and co-workers [24] and in agreement with Gazzano et al. [45], although amorphous silica is known to produce only transient inflammation compared to crystalline silica [7,46].

The generation of particle-derived free radicals in cell free environment, which is unrelated to hemolysis [20], appears not to play a role in IL-1β secretion for the present set of silica samples. Indeed, VS is less active in ˙OH release than Qz-1 and Qz-3. Moreover, neither VS nor Qz-3 are able to catalyze carbon-centered radicals, contrary to Qz-1 [41,47].

We showed that three factors were involved in the IL-1β release upon exposure to our silica samples, the ASC protein, phagocytosis and active lysosomal protease cathepsin B, ascertaining the implication of the inflammasome. Our results also suggest that lysosomal damage is required to activate the inflammasome. The ability of pristine and modified silica particles to induce the release of IL-1β in macrophages strictly paralleled their hemolytic activity (Figure 5), which depends on silica physico-chemical properties that could be modified by surface treatments. The correlation between the hemolytic potential of the silica particles and their IL-1β response suggests that the physico-chemical properties relevant for RBC membranolysis may also be implicated in the labilization of the phagolysosome and could mediate inflammasome activation. As previously reported [20], silanol distribution plays a central role in silica hemolytic activity. We could then speculate that the external RBC membrane and the internal phagolysosomal membrane could both have a structure sensitive to a specific silanol arrangement. Many decades ago the group of Wallingford et al. [48] noted that the physico-chemical reactivity of the RBC membrane may resemble the lysosomal one, based on the fact that agents which produce RBC lysis (Vitamin A, lysolecithin, weak acids, polyene antibiotics, and sodium urate crystals) also damage lysosomes. This seemed to be more evident for silica when both hemolysis and phagolysosome rupture were inhibited by the polymer PVPNO which is a strong hydrogen acceptor [38]. PVNO was recently reported by Peeters and co-workers [49] to reduce the level of Nalp3 inflammasome activation by quartz. Recent papers [50,51] reported a reduced *in vitro* and *in vivo* inflammasome activation following surface functionalization of multiwalled carbon nanotubes with the –COOH acidic group, which is largely deprotonated in physiological solution. A decrease in the inflammasome-dependent IL-1β production and in endosomal rupture was also evidenced by Morishige et al. [36] for amorphous silica particles after their modification with different functional groups (−COOH, −NH_2_, −SO_3_H, −CHO).

The peculiarity of silica is its strong hydrogen-bond potential compared to other inorganic compounds. This is mostly due to the intermediate electronegativity of silicon which falls between non metals oxides, acting as Brønsted acids, and metal oxides turning into hydroxo compounds with basic properties when hydrated. Through silanols, silica acts as a hydrogen donor in the formation of hydrogen-bond with hydrogen acceptors largely present at the biomembrane surface, including phosphate ester groups of phospholipids or secondary amide (peptide) groups of proteins [52].

## Conclusions

In this study we considered a panel of silica particles for investigating the connection between the hemolytic activity and the release of the pro-inflammatory cytokine IL-1β from macrophages. A strong correlation between hemolytic activity and pro-inflammatory potential was observed, suggesting that the same silica physico-chemical properties which are relevant in RBC membrane rupture may also be implicated in the labilization of the phagolysosome, leading to inflammasome activation. The present data strengthen the toxicological relevance of the hemolysis assay to predict the pro-inflammatory activity of silica dusts.

## Methods

### Silica samples

The five silica samples used, whose main characteristics are reported in Table 1, were: (*Qz-1*) the commercial microcrystalline α-quartz Min-U-Sil 5, largely used in studies of experimental silicosis and lung cancer [5], purchased from U.S. Silica Co. (Berkeley Springs, WV); (*Qz-2*) the Min-U-Sil 5 quartz heated in vacuum at 800°C for 2 h to reduce surface hydrophilicity [43,44,53]; (*Qz-3*) obtained by grinding a very pure quartz crystal from Madagascar in a planetary ball mill (Retsch S100, GmbH, Haan, Germany) for 3 h (70 rpm), then in the mixer mill (Retsch MM200) for 9 h (27 Hz). The grinding process was performed in an agate jar to keep silica free from impurities. This sample, contrary to what was found with other batches [20] of the same material, was inert in hemolysis. (*Qz-4*) obtained again from a pure quartz crystal from Madagascar following the same grinding procedure of Qz-3, and then treating 100 mg with a solution 0.1 M of hydrofluoric acid for 10 min. The dust was centrifuged (2500 rpm for 20 min), washed four times with distilled water and dried at 100°C for 3 h. The treatment was conducted with the aim to smoothen up surface defects and irregularities [44]. (*VS*) a vitreous silica with size distribution and surface area close to typical commercial quartz dusts, obtained by grinding a very pure silica glass (Suprasil) produced for optical applications in a ball mill (agate jar) for 3 hours (70 rpm).

### Chemical reagents

Dulbecco’s modified Eagle medium (DMEM), fetal bovine serum (FBS), phosphate buffered saline (PBS) and penicillin-streptomycin (10,000 U and 10,000 mg/ml) were obtained from Invitrogen (Bleiswijk, Netherlands). The WST-1 reagent was purchased from Roche Applied Science (Vilvoorde, Belgium). NaCl 0,9% was obtained from B. Braun Medical (Diegem, Belgium), Triton X-100 from Flucka (Buchs, Switzerland), the cathepsin B inhibitor CA-074-Me from Bachem (Switzerland). Methanol, dimethyl sulfoxide (DMSO), Tris buffered saline, Tween 20, lipopolysaccharide (LPS), cytochalasin D, ATP and chloroform were purchased from Sigma-Aldrich, 2-mercaptoethanol, Laemmli Sample Buffer from Bio-Rad (Hercules, USA) and hydrofluoric acid (HF) from Merck (Darmstadt, Germany).

### Particle characterization

Surface area measurements were performed by means of the BET method based on N_2_ adsorption at −196°C (Quantasorb, Quantachrome Instrument).

Particle size was obtained by using a flow particle image analyzer (Sysmex FPIA-3000, Malvern Instruments, UK; detection range: 0.8-300 μm). This instrument measures the diameter of the circle having the same projected area as the particle image detected. Measurements were carried out on sample suspensions at a concentration of 1 mg/ml in saline. Each sample was run at least four times with objective lens at 20× magnification in high power field (HPF) mode. The four analyses were then pooled to obtain the final mean value of size ± standard deviation (SD).

### Hemolysis of human RBCs

RBCs were separated from fresh human blood of a healthy volunteer donor not receiving any pharmacological treatment. The protocol for hemolysis measurement refers to Lu et al. [14], with minor modifications given in Pavan et al. [20]. Hemolytic activity of silica particles was evaluated on the basis of surface dose (cm^2^ silica/ml).

### Primary macrophage cell isolation and culture

Peritoneal macrophages were selected for the present study as they produce large amounts of IL-1β and can be easily obtained from genetically deficient animals. Macrophages were obtained by peritoneal lavage with 10 ml NaCl 0.9% of male C57BL/6 or ASC^−/−^ mice in a C57BL/6 background sacrificed with sodium pentobarbital. Mice were housed in positive pressure air-conditioned units (25°C, 50% relative humidity) on a 12 h light/dark cycle. ASC-deficient mice were obtained from the Transgenose Institute (Orléans, France).

Peritoneal lavage fluid was centrifuged for 10 min at 1250 rpm (Centrifuge 5804, Eppendorf, Hamburg, Germany), the supernatant was removed and cells (2 × 10^5^ cells/well) were seeded in 96-well plates using DMEM (1 g/l D-glucose) medium supplemented with 10% FBS, penicillin (100 U/ml) and streptomycin (100 μg/ml). Cells were incubated 4 h at 37°C in a 5% CO_2_-supplemented atmosphere.

### Cell priming and particle exposure

Macrophages were rinsed twice with cell culture medium and primed with LPS (100 ng/ml) for 18 h (37°C, 5% CO_2_).

Silica particles were heated at 200°C for 2 h just prior to suspension in order to sterilize them and inactivate any trace of endotoxin. Silica suspensions were prepared at the final concentration of 40 cm^2^ silica/ml in serum free DMEM. Suspensions were sonicated in a bath during 2 min. Serial dilutions were performed just before use to 20 and 10 cm^2^/ml. Silica suspensions or serum free DMEM (control) were distributed (200 μl/well) in six replicates in cell culture plates and incubated for 6 h. ATP (5 mM) (positive control) was added to parallel wells 1 h before the end of the incubation. In the experiments with inhibitors, LPS-primed macrophages were pre-incubated for 1 h with cytochalasin D (5 μM) or CA-074-Me (10 μM), the latter reconstituted in DMSO (in this case the negative control was serum free DMEM added with 0,1% DMSO). At the end of the exposure period, supernatants were collected in a new plate and stored at −20°C to assess IL-1β content, while cytotoxicity was determined on adhering cells.

### Cytotoxicity and Enzyme-Linked ImmunoSorbent Assay (ELISA)

Cytotoxicity was assessed with the WST-1 assay as described previously [54]. Briefly, WST-1 is a colorimetric assay quantifying mitochondrial activity as a measure of cell viability. 10% WST-1 reagent diluted in medium was added (100 μl/well) and culture plates were incubated about 40–50 min. Supernatants were moved to a new plate and absorbance of the formazan dye formed was measured at a wavelength of 450 nm and a correction wavelength of 690 nm by UV/vis spectrophotometry (Infinite 200, Tecan, Grödig, Austria). Results are expressed as a percentage of the negative control.

Culture supernatants were assayed for IL-1β using an ELISA kit for mouse IL-1β/IL-1 F2 (DY401, R&D Systems, Minneapolis, USA) according to the manufacturer’s instructions. This ELISA preferentially recognizes mature IL-1β, but also the pro-IL-1β form although to a lesser extent.

### Western blotting

Pro-IL-1β and mature IL-1β were discriminated by Western Blot. Primary macrophages were seeded at a density of 1.2 × 10^6^ cells/well in a 24-well plate with supplemented DMEM. Cell priming and particle exposure were as indicated previously, except that silica particles were tested at the single concentration of 20 cm^2^/ml. After exposure to particles, proteins in the supernatants were precipitated by adding an equal volume of methanol and 0.25 volume of chloroform. Supernatants were centrifuged for 15 min at 12,000 × g (Centrifuge 5804, Eppendorf). The upper alcoholic phase was discarded and a volume of methanol equal to the interphase containing the proteins and the lower chloroform phase was added. The mixture was centrifuged for 15 min at 12,000 × g. Supernatant was removed, the protein pellet dried for 10–15 min at 55°C and suspended in Sample Buffer (a mixture 1:20 of 2-mercaptoethanol and Laemmli Sample Buffer respectively). Alternatively, tissue culture pellets were lysed using 300 μl of Sample Buffer and, after thorough mixing, transferred to microcentrifuge tubes. Cell lysates and precipitates were stored at −20°C. After thawing, protein precipitates were sonicated for 5 min in a bath, boiled at 99°C for 5 min and centrifuged for 10 min at 14,000 rpm. Proteins were separated by 20% SDS-polyacrylamide gel electrophoresis (SDS-PAGE) (Mini-PROTEAN TGX 4-20%, BioRAD Life Science, Hercules, USA). A protein ladder was also added (PageRuler Plus Prestained Protein Ladder, Fermentas, St. Leon-Rot, Germany). Proteins separated by SDS-PAGE were transferred to a nitrocellulose membrane (Hybond-C Extra, Amersham Biosciences, Piscataway, USA). Before blocking, Ponceau-staining was used to control protein levels. The membrane was blocked with 5% milk for 1 h at room temperature (RT) and then incubated with the primary antibody (polyclonal goat anti-mouse IL-1β IgG, AF-401-NA, R&D Systems, Minneapolis, USA) overnight at 4°C on a rotating platform. The membrane was washed three times with Tris Buffered Saline containing 0.1% Tween 20 and incubated for 1 h at RT with the respective secondary horseradish peroxidase-conjugated antibody (rabbit anti-goat IgG-HRP, Santa Cruz Biotechnology, Santa Cruz, USA). After washing the membrane three times with Tris Buffered Saline containing 0.1% Tween 20 and once with Tris alone, the blot was developed using the SuperSignal West Pico or Femto chemiluminescent substrates (Thermo Scientific, Rockford, USA) according to the manufacturer’s instructions.

### Statistical analysis

Data are presented as mean ± standard deviation (SD). Differences between groups were analyzed by one-way analysis of variance (ANOVA) with post hoc Tukey’s pairwise comparison test. Differences with *p* value < 0.05 were considered statistically significant. Linear regression analysis (Pearson’s coefficient) was applied in Figure 5.

## Additional file

Additional file 1.
**Figure S1.** Cytotoxic activity caused by increasing doses of different silica particles in primary murine macrophages. **Figure S2.** IL-1β release from primary murine macrophages induced by increasing doses of different silica particles.
