# Supplementary material for: Why does the hemolytic activity of silica predict its pro-inflammatory activity?
Source: Part Fibre Toxicol. 2014 Dec 19;11:76. doi: 10.1186/s12989-014-0076-y (PMC4318150; doi:10.1186/s12989-014-0076-y)
Supplement: Additional file 1. — Figure S1. Cytotoxic activity caused by increasing doses of different silica particles in primary murine macrophages. Figure S2. IL-1β release from primary murine macrophages induced by increasing doses of different silica particles. [file 12989_2014_76_MOESM1_ESM.pdf]

Additional file 1

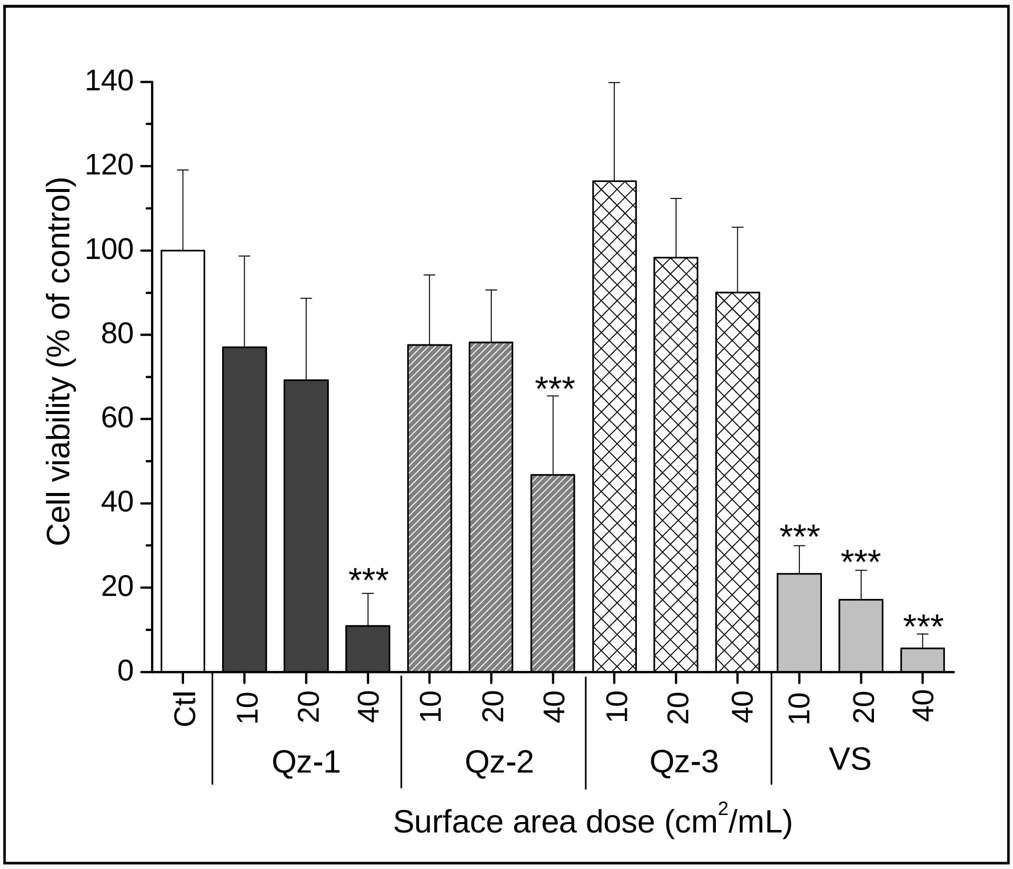

Figure S1. Cytotoxic activity caused by increasing doses of different silica particles in primary murine macrophages.

LPS-primed primary murine macrophages were incubated with increasing doses (10, 20 and 40 cm^2/ml) of silica for 6 h and then evaluated for cell viability by means of the WST-1 assay. The silica samples included a commercial quartz (Qz-1), the same quartz heated at 800° C (Qz-2), a pure quartz (Qz-3) and a vitreous silica (VS). Results are expressed as percentage of the control (macrophages not exposed to silica particles - Ctl). Data from one representative experiment performed in six replicates are showed and expressed as the mean ± SD. *** $p < 0.001$ *vs* control not exposed to silica particles.

The silica particles considered here showed different levels of cytotoxic activity but all with a dose-effect relationship, except Qz-3 for which the difference compared to the control is not statistically relevant at any dose investigated.

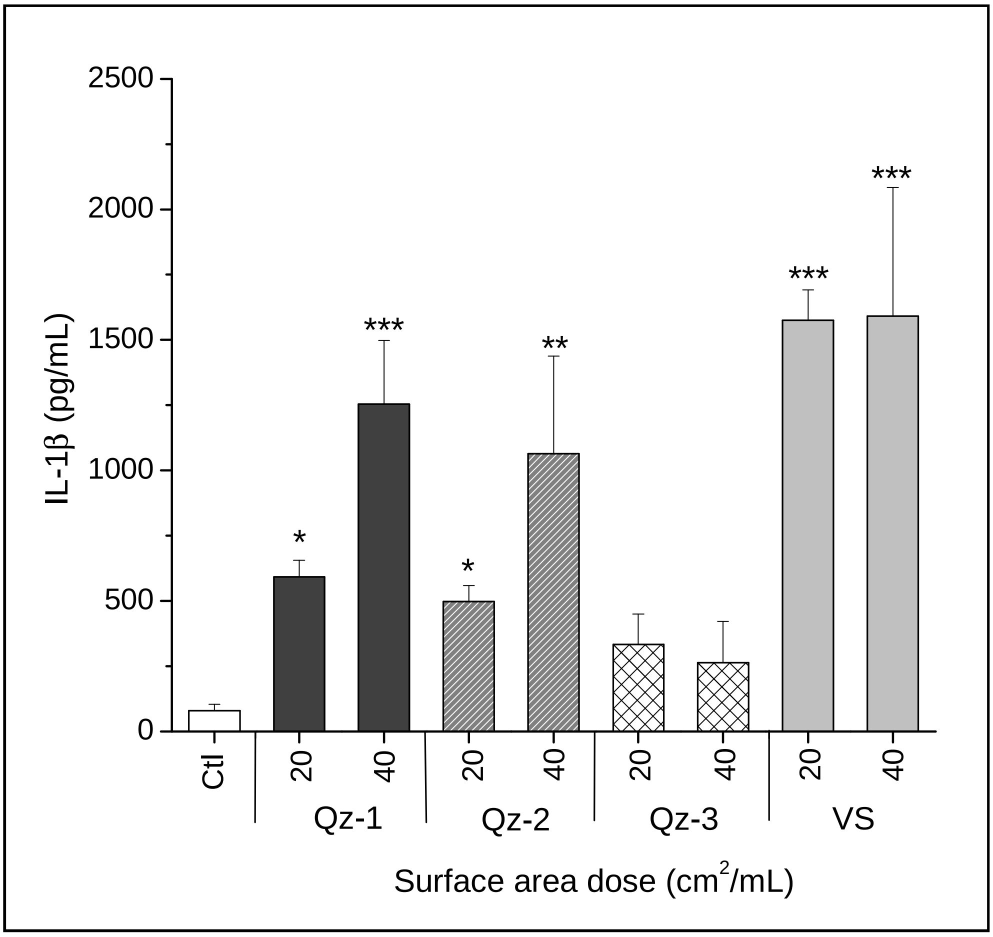

Figure S2. IL-1β release from primary murine macrophages induced by increasing doses of different silica particles.

LPS-primed primary murine macrophages were incubated with increasing doses of silica (20 and 40 cm^2/ml) for 6 h and then evaluated for IL-1β production (pg/ml) in culture supernatants by ELISA. The silica samples were a commercial quartz (Qz-1), the same quartz heated at 800° C (Qz-2), a pure quartz (Qz-3) and a vitreous silica (VS). Data from one representative experiment performed in five replicates are showed and expressed as the mean ± SD. *$p < 0.05$, **$p < 0.01$ and *** $p < 0.001$ *vs* control not exposed to silica particles.

Qz-1 and Qz-2 showed an increased IL-1β release by increasing the particle dose from 20 to 40 cm^2/ml. Qz-3 was not statistically different compared to the control at both the doses investigated. VS was the most active compared to the other silica particles, already from the lowest dose.
